# Dynamic Deformation in Nuclear Graphite and Underlying Mechanisms

**DOI:** 10.3390/ma17184530

**Published:** 2024-09-14

**Authors:** Melonie Thomas, Hajin Oh, Ryan Schoell, Stephen House, Miguel Crespillo, Khalid Hattar, William Windes, Aman Haque

**Affiliations:** 1 Department of Mechanical Engineering, The Pennsylvania State University, University Park, PA 16802, USA; melonie.thms@gmail.com (M.T.); oh339@purdue.edu (H.O.); 2 Center for Integrated Nanotechnologies, Sandia National Laboratories, Albuquerque, NM 87185, USA; rmschoe@sandia.gov (R.S.); sdhouse@sandia.gov (S.H.); 3 Department of Nuclear Engineering, University of Tennessee, Knoxville, TN 37996, USA; mcrespil@utk.edu (M.C.); khattar@utk.edu (K.H.); 4 Idaho National Laboratory, Idaho Falls, ID 83415, USA; william.windes@inl.gov

**Keywords:** nuclear graphite, heavy ion irradiation, creep deformation, slip or ripplocation bands, in situ transmission electron microscopy

## Abstract

Time-dependent deformation in nuclear graphite is influenced by the creation and migration of radiation-induced defects in the reactor environment. This study investigates the role of pre-existing defects such as point defect clusters and Mrozowski cracks in nuclear graphite IG-110. Separate specimens were irradiated with a 2.8 MeV Au^2+^ beam with a fluence of 4.38 × 10^14^ cm^−2^ and an 8 MeV C^2+^ beam with a fluence of 1.24 × 10^16^ cm^−2^. Microscopic specimens were either mechanically loaded inside a transmission electron microscope (TEM) or subjected to ex situ indentation-based creep loading. In situ TEM tests showed significant plasticity in regions highly localized around the Mrozowski cracks, resembling slip or ripplocation bands. Slip bands were also seen near regions without pre-existing defects but at very high stresses. Ex situ self-ion irradiation embrittled the specimens and decreased the creep displacement and rate, while heavy ion irradiation resulted in the opposite behavior. We hypothesize that the large-sized gold ions (compared to the carbon atoms) induced interplanar swelling as well as cross-plane channels for increased defect mobility. These findings illustrate the role of pre-existing defects in the dynamic relaxation of stresses during irradiation and the need for more studies into the radiation environment’s impact on the mechanical response of nuclear graphite.

## 1. Introduction

Graphite is a critical nuclear material because of its excellent chemical and thermal stability. However, the structural reliability of the material is also important since the material is inherently brittle. The ability to creep or relax the stress built in by external thermo-mechanical loads and intense neutron radiation is critical in mitigating the structural degradation of graphite. At the same time, monitoring creep over the reactor lifetime is necessary because dimensional changes impact the reactor operation and safety [1]. Accordingly, the creep of graphite has been studied since the beginning of the nuclear reactor era and it will continue to be relevant in current gas-cooled reactors, where it serves as a moderator and structural material [2]. Macroscopically, creep manifests as gradual and time-dependent deformation under constant stress. Like in other engineering materials, the process begins with instantaneous elastic strain followed by transient creep and then steady-state creep. The magnitudes of the elastic and transient strain are approximately equal [3]. Neutron fluence and temperature strongly influence the steady-state creep coefficient [4,5]. Another influential parameter is the degree of anisotropy in the material, which dictates the dynamics of both the formation and migration of the defects [6].

The motivation for this study comes from the gap between the macroscopic properties (such as creep) and microscopic deformation fundamentals in nuclear graphite. Mathematical visco-elastic creep models are available in the literature that (a) accommodate structural and radiolytic oxidation, such as the UK creep model; (b) neglect changes in the coefficient of thermal expansion and add recoverable creep, such as M2 model; and (c) accommodate dimensional change, such as the Kelly Burchell model and thermally activated creep [7]. However, irradiation changes most parameters (such as the coefficient of thermal expansion, secondary creep coefficient, Young’s modulus, and percentage volume change) that are inputs to these models. These parameters depend on the constituent materials and the manufacturing processes [8,9]. For a fundamental understanding of the creep properties, it is important to examine the microstructure and defects [10]. This involves the entire spectrum of defects—from atomic-scale vacancies to the micron-scale pores and grains consisting of the binder and filler phases.

This study aims to examine nanoscale features such as crystallites, interfaces, and Mrozowski cracks as they are mechanically loaded inside the transmission electron microscope (TEM). The underlying theme is that interfaces and cracks can influence time-dependent deformation in materials by modifying the localized stress distribution and energy dissipation [11]. The abundance of interfaces at the nanoscale is evident from the nanoscale characteristic size of the crystallites [12]. The nanoscale Mrozowski cracks [13] are also abundant, since they originate from the differential thermal expansion in the anisotropic solid. These effects are expected to be intensified by high temperatures and irradiation. We used ion irradiation as a surrogate for neutron irradiation. Ion irradiation allows for the comprehensive design of experiments with well-controlled parameters such as energy, dose rate, and temperature. The major advantage is the rapid accumulation of end-of-life doses in short periods and the immediate availability of nonradioactive samples, which is impractical for neutron irradiation. These benefits come with other challenges to be considered, including the high damage rate and the small volume of material that can be irradiated [14].

Nuclear graphite is a brittle material; therefore, microscale mechanisms for large-scale plasticity are limited. The classical perspective is based on point defects and dislocations generated by neutron radiation. Additionally, the hexagonal close-packed (HCP) nature of the system makes most of these defects mobile in the basal planes [15]. However, interstitials and prismatic dislocations can be mobile in the other planes as well. It is suggested that irradiation increases sp2-hybridized defects, and dislocation-mediated amorphization overshadows point defect-based mechanisms [16]. More recent observations have suggested new mechanisms such as ruck/tuck [17] or ripplocations [18], which have been theoretically and experimentally discussed in the literature. These fundamental mechanisms are very commonly observed in nuclear graphite and can have a strong influence on creep deformation. Often overlooked is the predominance of interfaces in nuclear graphite and the associated impact on mechanical properties. Specifically, interfaces can greatly increase diffusional creep and energy dissipation in solids, particularly at high temperatures [19]. For nuclear graphite, the interfaces could be filler–binder or more predominantly crystallite–crystallite. At a relatively large length scale, gas-filled pores and voids can influence viscoelastic deformation, a mechanism that is very relevant for graphite [20]. As instrumentation permits more detailed analysis, it is interesting that the formation of kink or ripplocation bands is appearing more frequently in the literature [18,21,22]. Such deformation bands are strong indications of extensive but highly localized deformation fields.

For comparison, the literature on the bulk behavior of graphite is rich because of the indispensable nature of the material for gas-cooled reactors. However, the multi-scale nature of the defects has also led to microscopy and spectroscopy studies to examine the nano- to atomic-scale defects and microstructures. However, most of these studies are postmortem observations only. Our recent study demonstrated the advantages of in situ mechanical testing inside the transmission electron microscope (TEM) [21]. Although this study did not investigate time-dependent deformation, it highlights the role of real-time visualization. One could envision an ideal experiment that involves in situ ion irradiation, high temperature, and quantitative mechanical testing to directly visualize defect morphology changes under irradiation and mechanical loading at very high temperatures. This early in situ experiment takes a first step by exploring in situ creep testing in the TEM of a pristine and two previously ion-irradiated specimen sets.

## 2. Materials and Methods

**Figure 1:**
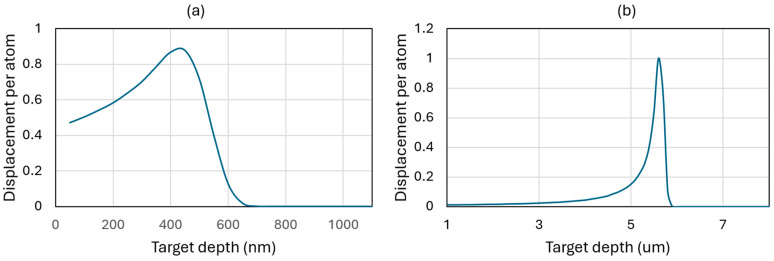
Stopping and Range of Ions in Matter simulation results for the displacement per atom (dpa) in (**a**) Au^2+^- and (**b**) C^2+^-ion-irradiated specimens.

Nuclear graphite IG-110 (Toyo Tanso Co., Kagawa, Japan) was Au-ion irradiated at the Ion Beam Laboratory at Sandia National Laboratories using the 6 MV HVEE tandem accelerator and C-ion irradiated at the Tennessee Ion Beam Materials Laboratory at the University of Tennessee Knoxville using the 3 MV NEC tandem accelerator. Adjustable beam slits were used to define the irradiation areas. The ion beam was defocused and rastered in the horizontal and vertical directions with the aim of producing a region that was homogeneously irradiated. Beam homogeneity was verified by ion-induced luminescence from quartz targets and was found to be within 10% throughout the irradiated area [23]. All ion irradiation experiments were performed at nominal room temperature. The graphite sample was a fine-grain (average grain size: 20 μm) graphite that is well-studied in the literature for high-temperature gas-cooled reactors. The bulk specimen was irradiated with two types of ions at 25 °C, (a) a 2.8 MeV Au^2+^ beam with a fluence of 4.38 × 10^14^ cm^−2^ and (b) an 8 MeV C^2+^ beam with a fluence of 1.24 × 10^16^ cm^−2^. The carbon ion beam was normal to the surface of the sample prior to FIB work. The Au ion beam was at 60° degree to the side of the already-prepared pillars. The ion energies and fluences were chosen with the aid of irradiation damage simulations using the Stopping and Range of Ions in Matter (SRIM) code in “Ion Distribution and Quick Calculation of Damage” mode. For the 2.8 MeV Au beam, the displacement energy of carbon atoms and the surface binding energy for sputtering were set to 28 eV and 7.4 eV, respectively. Figure 1 shows SRIM simulations of both conditions.

**Figure 2:**
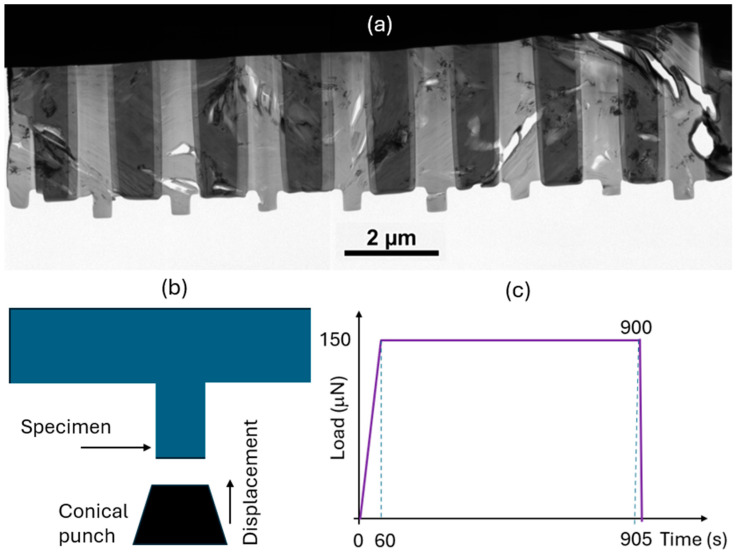
(**a**) Focused-ion-beam-milled specimens for in situ TEM mechanical testing. (**b**) Schematic diagram of a conical-punch-based micro-pillar specimen compression. (**c**) An example of a time-dependent deformation test at constant force.

Both pristine and irradiated bulk graphite samples were milled with a focused ion beam (FIB) using a Scios 2 Dual Beam scanning electron microscope (Thermo Fisher Scientific, Waltham, MA, USA) to prepare electron-transparent specimens. The individual specimens were cuboid shaped with dimensions of approximately 450 nm tall, 400 nm wide, and 150 nm in thickness. This geometry provided a balance between the electron transparency of the specimen to detect any defects and the probability of instability by buckling or bending upon mechanical loading. A detailed description of the micro-pillar preparation is discussed in the Supplementary Information. Figure 2a shows a low-magnification TEM image of the representative specimens.

### 2.1. In Situ Experiments

**Figure 4:**
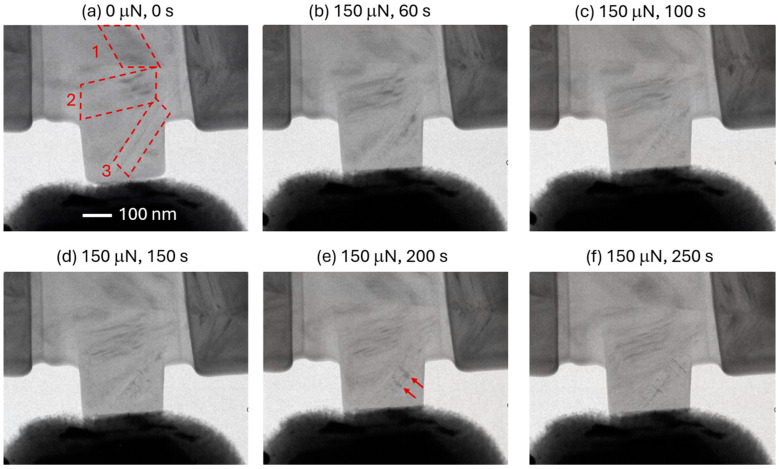
Snapshots taken from in situ TEM dynamic micro-pillar compression tests on pristine IG-110 nuclear graphite. (**a**) Unloaded specimen showing three deformation bands identified with dashed paralellograms. (**b**) Loaded to 150 μN in 60 s. (**c**–**f**) Images acquired at 100, 150, 200, and 250 s of loading at a constant value of 150 μN. Arrows in Figure 4e show extensive cross-slip.

**Figure 6:**
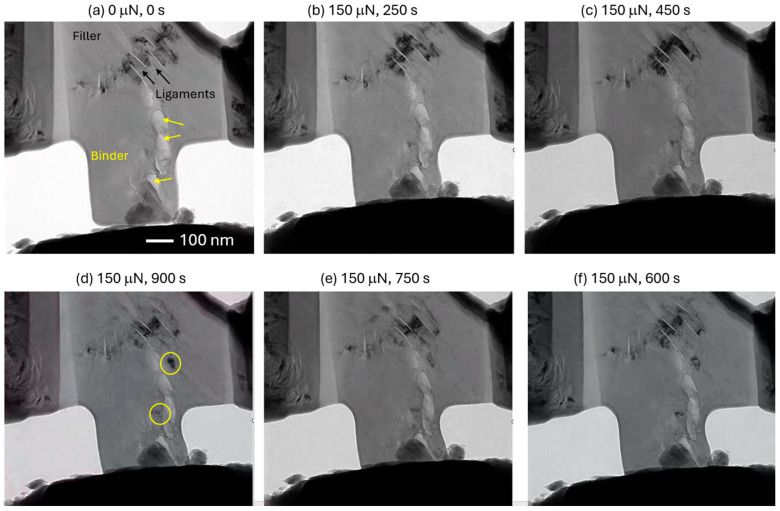
Snapshots taken from in situ TEM dynamic micro-pillar compression tests on 2.8 MeV Au^2+^-ion-irradiated IG-110 nuclear graphite. (**a**) Unloaded specimen showing filler-binder interface with yellow arrows and deformation ligaments with black arrows. (**b**) Loaded to 150 μN in 60 s. (**c**–**f**) Constant load maintained over time. Yellow circles in Figure 6d indicate areas with newly formed deformation bands.

**Figure 7:**
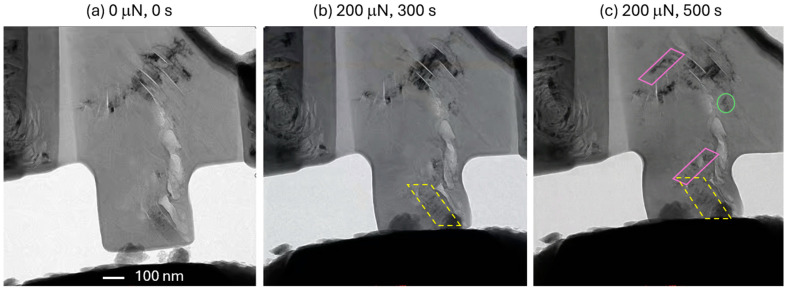
Repeated experiment on Au^2+^-ion-irradiated IG-110 nuclear graphite at higher load. (**a**) Before loading, (**b**) after 300 s at 200 μN load, showing a newly developed deformation band with the dashed line parallelogram and (**c**) after 500 s at the same load, showing two new cross-slip deformation bands with solid line parallelograms. The circle denotes active deformation but without a prismatic band structure.

The experiments were performed inside a mono-chromated FEI Titan G2 environmental transmission electron microscope (ETEM) at 300 kV (Thermo Fisher Scientific, Waltham, MA, USA). It is well known that graphite specimens are damaged by electron irradiation during scanning TEM mode. Even though we did not perform STEM for the in situ mechanical testing, we avoided long exposure at higher magnifications, as shown in Figure 4, Figure 6 and Figure 7. In addition, the acquired TEM images were examined for other possible effects of electronic irradiation such as localized amorphization. Load and displacement were applied and measured using the Bruker/Hysitron PI-95 TEM Picoindenter system (Bruker/Hysitron, Eden Prairie, MN, USA). Figure 2b,c schematically shows the time-dependent test protocol, where the loading and unloading times were varied from 60 to 300 s (depending on the specimen) to acquire stable data. The constant load hold time was 900 s. However, many of the specimens, particularly pristine ones, failed prematurely. The PI-95 1 µm conical flat punch was used for all compression tests, and the tests were run in displacement-controlled mode by setting the displacement depth to half of the initial height of each pillar (the displacement rate was set to ~0.5 nm/s). The indenter system is displacement controlled but allows the user to maintain a constant force, as reflected in Figure 2c. Load–displacement measurements and TEM video of microstructural changes were recorded for all static and dynamic tests. We also performed standard compression tests with no hold time.

### 2.2. Ex Situ Experiments

**Figure 3:**
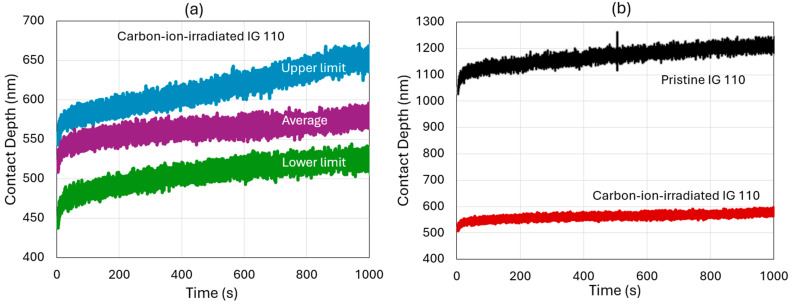
(**a**) Contact depth vs. time data showing the lower, average, and upper limits of the load–displacement response in carbon-irradiated IG-110 graphite. (**b**) Average data trend for pristine and carbon-irradiated specimens.

To quantify the time-dependent behavior of the specimens under different irradiation conditions, we performed nanoindentation creep experiments. This localized probing technique requires a careful approach and interpretation because of the heterogeneity in both the material composition and the defect sizes [24]. Indentation locations were chosen carefully under the microscope to avoid defects on the surface. To minimize the error, the region of interest consisted of 9 different regions, each region with 3 × 3 array of indentation spots. The indents were made using a Hysitron TI 980 system (Bruker, Billerica, MA, USA) with a Berkovich tip. The loading, hold, and unloading times were 0.5, 1000, and 0.5 s, respectively, with a peak load of 12.5 mN. Figure 3a shows the maximum spread in the raw data for three regions (shown by three different colors) of interest in the carbon-ion-irradiated specimen. The very large spread (about 18.50%) is characteristic of localized probing as well as material heterogeneity. Nevertheless, we argue that the technique is helpful in distinguishing the material properties if the average values are significantly larger than the spread. This is shown in Figure 3b, where we plot the average trends of the pristine and carbon-ion-irradiated specimens. The amount of hardening due to radiation is significant compared to the data spread observed in the study.

## 3. Results

In situ TEM deformation experiments at constant load were performed on both pristine and 2.8 MeV Au^2+^-ion-irradiated graphite specimens. Figure 4a shows the pristine specimen in the unloaded condition. Nuclear graphite is a brittle material, but we could identify three deformation bands (marked numerically with dashed parallelogram in Figure 4a) resembling slip bands in metals in the specimen. These are also proposed to be ripplocation bands in the literature. These bands were seen in most other specimens evaluated in this study (pristine and irradiated), despite varying in their location and numbers. Unfortunately, this aspect of the specimens makes them predictably different from each other, even with same physical dimension and applied load. Nevertheless, this study highlights the significance of these deformation bands on the mechanical properties. Even at this low magnification, the slip activities in these bands were very prominent. As shown later, the contrast in these bands come from defects (such as large clusters of vacancies). Interestingly, one would expect the band marked 3 to be the most active because it has the smallest cross-section in that region. However, the loading phase showed slip activities marked by discernible changes in contrast in all three systems.

The pristine specimens did not survive more than 250 s of constant loading at 150 μN. The amount of hold time until catastrophic failure varied from 100 to 250 s for the set of specimens, which could be due to the difference in the number and orientation of the deformation bands. In addition, there could be discrepancies in the actual specimen thickness because of the nature of the FIB milling process. Nevertheless, the TEM experiments clearly show that these bands are the only sources of time-dependent deformation at constant load. A video clip of this experiment is available as Supplementary Information. The careful examination of Figure 4c–f reveals highly localized deformation in the band systems 2 and 3. In particular, deformation band 3 showed evidence of extensive cross-slip (shown by the arrows in Figure 4e). Cross-slip plasticity is mostly seen in high-symmetry crystals [25], where dislocations move from one slip plane to another. The activation of multiple slip systems essentially multiplies the dislocation density. Therefore, cross-slip plasticity is more effective than self-replication (for example Frank–Read type) in a single-slip system [26]. Hexagonal closed-packed systems lack symmetry; therefore, dislocations mostly move or slip on specific crystallographic planes and directions. The primary slip system in HCP crystals is the basal planes, but very high shear stress or temperature can induce cross-slip. Room-temperature cross-slip plasticity in graphite is yet to be reported, but for metals, this can take place in the prismatic or pyramidal planes [27]. However, it could be possible because of the very high stress and the abundance of ultra-sharp stress concentrations through the Mrozowski cracks.

**Figure 5:**
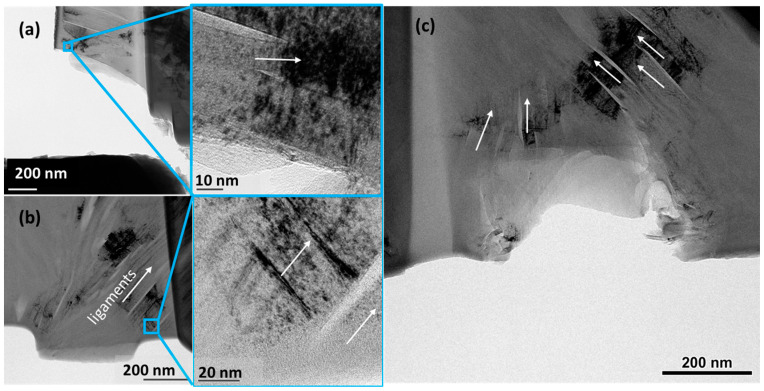
(**a**–**c**) TEM micrographs of a collection of buckled ligaments separated by nanoscale cracks showing extensive dislocation and point defect clusters. Ligaments are shown in white arrows.

A unique observation in the in situ TEM creep experiments is the evidence of defects escaping the specimen instead of being accumulated. The Supplementary Video associated with Figure 4b–f shows defects moving through the deformation bands and without piling up as expected. We suggest that this is due to the specimen thickness, which allows the defects to escape to the surface. The implication is that the deformation bands are excellent sources of defects, which can be explained by their abundance near the nanoscale Mrozowski cracks that are often separated by the ligaments that function as nanoscale deformation bands. This is evident from Figure 5 below, where the defects arise from the abundant and sharp crack tips. This conjecture is well supported by the literature, where the ligaments between two cracks are always seen to bend, buckle, and twist, while crack-free regions do not show any signs of plastic deformation.

To study the effect of ion irradiation on creep deformation, we performed in situ TEM experiments on the specimens. Figure 6 shows load and time history for a specimen, showing significantly longer time under constant load before failure. This specimen contained microscopic features typically seen in filler and binder regions [28]. The filler region shows nanoscale cracks and bridging ligaments, which showed very high defect density in these regions. During loading, extensive defect generation and mobility were observed in the ligaments (space between two parallel cracks). For both pristine and irradiated specimens, the ligaments contained very high defect density, suggesting them to be potent multiplication sources. Figure 5 suggests the existence of large clusters of point defects. The literature has ample evidence (atomic-scale imaging) of dislocations and ruck/tuck or ripplocations that are active or mobile in a direction parallel to the cracks [18,21,22]. Figure 6b–f shows extensive motion such as dislocation and point defects at a constant load of 150 μN. It is important to note that the ligament plasticity does not propagate through the specimen—either because it is a highly localized phenomenon or because it is easier for them to escape through the surface. However, through the period, we observed activation in some locations that have no ligaments. Examples of these sources are shown by yellow circles in Figure 6d. These locations continue to generate the dislocations and, like the ligaments, expel them through the surface. A video clip showing these events is available as a Supplementary Material. We propose that the generation and motion of these dislocations, albeit localized, offer a pathway toward the dissipation of the energy, which allows the irradiated specimens to withstand the applied external load for a longer time compared to the pristine specimens. The longest time the pristine specimens survived the loading was 250 s. This is anomalous to the evidence given in the literature, where irradiation is shown to harden the material and decrease its ability to sustain the load [29].

Since the 2.8 MeV Au^2+^-irradiated specimens withstood the 150 μN load for the specified 900 s, they were subjected to new set of experiments with a 200 μN load. The results are shown in Figure 7. The high load initiated dislocation motion in the previously observed ligament locations. But more importantly, a new deformation band (marked with yellow dashed parallelogram in Figure 7b) became activated. Because of the higher external stress and the stress concentration at the crack tips, we observed cross-slip in two new deformation bands, shown in solid line parallelograms in Figure 7c. Another location is shown in the circle, where the deformation band is not prismatic but became active over time. These observations are remarkable because the applied load is constant, but the stress field is highly dynamic with the generation of new defects, localized deformation in the bands, and the stress concentrations around the cracks and pre-existing defects.

## 4. Discussion

Combining in situ ion irradiation and nanomechanical testing offers the advantage of providing direct insight into the deformation and failure mechanisms of irradiated materials like nuclear-grade graphite. It creates many challenges and potential artifacts associated with the electron beam exposure, vacuum conditions, and small-scale geometries. This discussion will detail each and discuss controls performed to address those potential challenges in this study and future experiments to explore other potential nuances of this current study.

**Figure 8:**
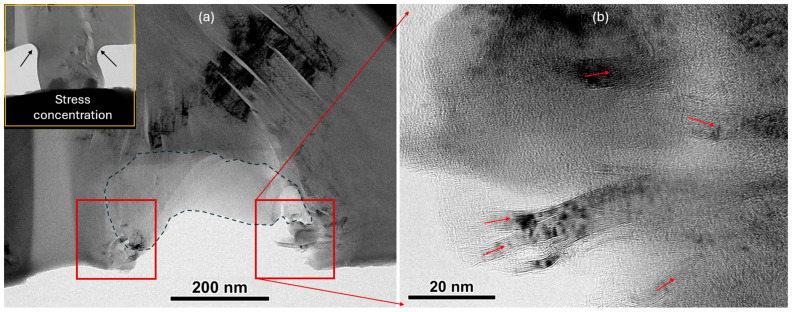
(**a**,**b**) Fracture surface showing failure by creep deformation at the base of the micro-pillar specimen. Inset shows the specimen before the fracture with arrows indicating the stress concentration.

A specific issue with graphite imaging in TEM is the risk of electron irradiation. Since the creep experiments involve significant electron beam exposure times, we performed them at very low magnifications to minimize electron dose while allowing for real-time observation. Unfortunately, this prevented us from investigating the atomic-scale to nanoscale deformation mechanisms. Despite this limitation, dislocation bands are observable and highly active in the regions marked by parallelograms in Figure 7. When needed, high magnification was utilized to observe the highly localized plasticity that originated and migrated through the ligaments, as defined by the distance between two nanoscale cracks (Figure 6a). These plastic deformation events are triggered by very high stress or local inhomogeneities and are presumed to not be as heavily impacted by electron beam artifacts. By utilizing higher magnification, we could examine the fracture surfaces of these specimens to extrapolate the fundamental mechanisms. Figure 8 shows the fracture surface of the specimen after 500 s of creep loading at 200 mN. The inset shows the specimen right before the catastrophic fracture. The two arrows indicate the location of stress concentration in the specimen, which shows significant amount of bulging due to the plastic deformation. After fracture, both these locations are marked with the squares shown in Figure 8a. Figure 8b shows one of the stress-concentrated regions at higher magnification. It is obvious that there are deformation bands (marked with arrows) created by extremely high stress concentrations because these deformation bands were not present before the experiment. These deformation bands originate from extremely high stress to help accommodate growth to widths ranging from a few nm to tens of nm. We suggest based on these observations and the relevant literature that the fundamental mechanism for the formation of these bands is stress-induced graphitization [30,31], a localized phase transformation into well-aligned bands of crystalline graphite while the surroundings remain turbostratic. It is possible that the terminal crack nucleated after the failure of these deformation bands. As soon as that happens, the specimen was unable to bear the external load, and fractures propagated catastrophically through the base of the micro-pillar specimen marked with the dashed line in Figure 8a. The fracture surface appeared to be smooth, indicating the separation of basal planes and not the plastic tearing of deformation bands, as in Figure 8b. This is possible because of the layered nature of the graphite material. While the power of the in situ quantitative TEM experiments is directly correlated with the active mechanisms of the nanoscale load and displacement, revealing the deformation and failure mechanisms, it is limited by the inherently heterogenous and small-scale nature of FIB-produced, nuclear-grade graphite pillars.

**Figure 9:**
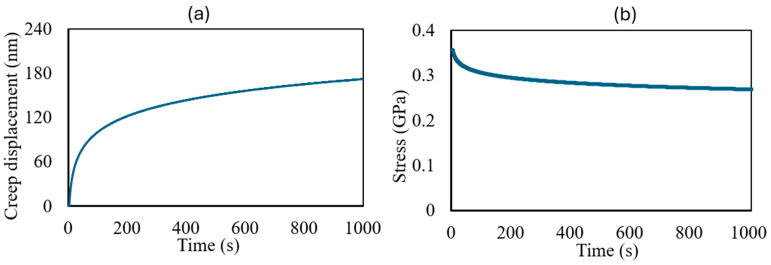
Ex situ indentation creep results for (**a**) creep displacement and (**b**) stress rate over time while the load is kept constant.

In an attempt to overcome the small-scale and unique geometries of the in situ ion irradiation of a bulk specimen heterogeneous in material composition as well as the size and aspect ratio of the pre-existing defects, we performed ex situ studies to compliment the in situ experiments. To gain quantitative understanding, we performed ex situ nanoindentation creep experiments on pristine, 2.8 MeV Au^2+^-, and 8 MeV C^2+^-ion-irradiated specimens. The advantage of these experiments is that one can quickly measure the fundamental parameters like creep displacement and strain/stress rates. However, a challenge arises from the inherently localized probing of the mechanical properties of a heterogenous material like nuclear graphite. Therefore, a significant number of indentations needs to be performed and averaged to extract the trend in property information, something that is not possible during the in situ TEM experiments. For the pristine IG-110 graphite, these average parameters are shown in Figure 9.

**Figure 10:**
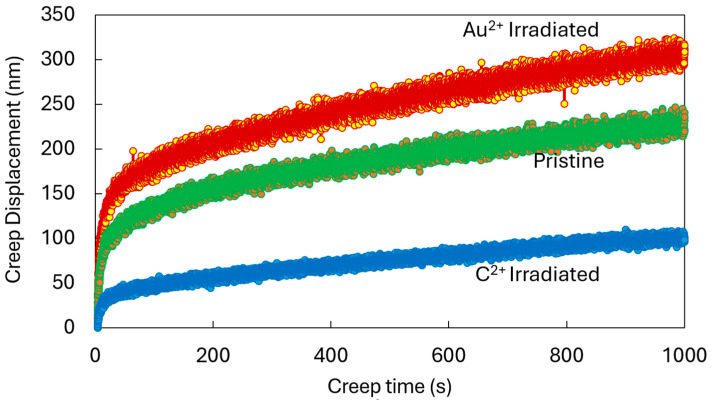
Ex situ indentation creep displacements at constant load for pristine and ion irradiated specimens.

Figure 10 shows our ex situ experimental results for the ion irradiation effects on creep deformation. The carbon-ion-irradiated specimens show the lowest primary and secondary creep rates. This is expected from the literature, where radiation-induced hardening is commonly ascribed to radiation hardening [32]. The interlayer spacing increases with the irradiation due to the clustering of vacancies, which accumulate between basal planes. This leads to the increase in lattice parameters and d-spacing in the c-direction [33]. The average hardness increases from 0.4 GPa to 0.93 GPa, while the indentation creep rate decreases from 28.5% to 23.5%. The indentation creep rate is obtained by dividing the maximum displacement during the loading stage by the creep displacement during the holding stage. Therefore, the decrease in this value reflects less creep deformation. Interestingly, the irradiation response to the gold ions was the opposite. Our previous static experiments explored the fundamental mechanisms behind this, where we observed an abundance of deformation bands leading to large plasticity [21]. Our hypothesis is that the very large size of the 2.8 MeV Au^2+^ atoms not only creates vacancy clusters but also facilitates the cross-plane motion of these defects. In comparison, the defects created by the 8 MeV C^2+^ ions are significantly smaller clusters that are primarily restricted to the basal planes. To the best of our knowledge, there is no systematic study on the effects of ion size, energy, displacement per ion, and nuclear/electronic stopping on the radiation response and subsequent mechanical property evolution of nuclear graphite. It is important to note that the highly localized probing in nanoindentation may not be directly applicable to modeling by classical creep models suitable for bulk materials but does provide insight into the global mechanical response to the nuances of the irradiation environment.

More generally, this study focused on the fundamental mechanisms behind creep deformation of IG-110 nuclear graphite. While the literature has theoretical models and experimental data on the bulk creep deformation of nuclear graphite, the findings are mostly quantitative, with some postmortem evidence of the microstructure and defects. The uniqueness of our in situ TEM approach is to combine a mechanical loading scheme with the simultaneous acquisition of qualitative microstructural visualization. We also performed ex situ nanoindentation creep experiments to investigate general agreements in the findings.

An important finding of this study is that even though nuclear graphite is brittle in the bulk form, some pre-existing defect structures may allow for highly localized plasticity. This study identifies two basic types of defect structures that allow for plasticity. The first one is the region containing nanoscale Mrozowski cracks, where the ligament between two cracks becomes the source for dislocations. In our in situ TEM creep experiments, we found these regions to be very sensitive to the applied stress because of their activity levels even when the external load was kept constant. The reason behind their sensitivity to the applied load is because the ligaments have significantly higher values of buckling or bending compliance, and they are also adjacent to the crack tips for stress concentration. The second types of defect structures appeared in the solid parts of the specimens and were not related to the pre-existing cracks. We suggest that these are regions that experience very high stress (especially if concentrated by specimen geometry or pre-existing features). The fundamental mechanism behind this could be stress-induced graphitization. In both cases, the TEM evidence suggested plasticity in parallelogram shaped shear bands. A shortcoming of this study is the lack of direct HRTEM-based evidence of these proposed deformation mechanisms. A more comprehensive model would consider other microstructural features such as quinoline-insoluble (QI) particles (Figure 7). QI particles are byproducts of coal tar processing that evolve into densified spheres upon irradiation. The bonding of QI matter with the surroundings is weak, which may alter both localized deformation and overall dimensional shrinkage [34].

Another finding of this study is the difference in irradiation hardening caused by variation in the radiation environment. Although further work is needed to fully elucidate the governing factors, it is hypothesized that when the cluster size created by the ion strike is small, the bulk material shows signs of hardening. This is commonly observed in a wide class of materials and is ascribed to amorphization or increases in disorder, limiting planar slip. We observed this behavior for 8 MeV self-ion irradiation. However, gold ions, which are significantly larger ions that create more damage, produced the opposite effect. The higher levels of creep and plasticity exhibited by 2.8 MeV Au^2+^-ion-irradiated specimens could be because of large clusters of defects that create interplanar swelling or other multiplanar damage that allows defects to be mobile in both basal and nondominant cross-slip planes. The term irradiation softening appears in the literature [35] but is not common for graphite materials. Despite this, the basic mechanism attributed to radiation softening could essentially be the same in graphite. In this softening mechanism, it is assumed that an excess concentration of radiation point defects (vacancies and interstitials) may enhance diffusional plasticity to soften the material [36]. A proper analysis would require a controlled set of experiments not only with different cascade sizes but also with various ion species, energies, fluxes, and fluences because all of these parameters can influence mechanical softening. It is also important that HRTEM imaging mode is used to unambiguously detect the defects and their types. In this study, we used high magnification (Figure 5 and Figure 8) to identify the defects. However, there still could be room for misinterpretations. Future efforts should address this shortcoming.

Finally, the importance of experimental design cannot be overstated for nuclear graphite materials. The inherent heterogeneity in material composition and defects (size, type, length scale) implies that a single length-scale investigation may not be able to explain the radiation response of the material. The experimental results presented here should not be directly extrapolated or quantitatively compared to the bulk scale. This is because the specimen preparation itself is challenging and may cause variation in measured properties. Another deviation from the realistic environment of a nuclear reactor is the discrepancy between ion and neutron irradiation in nuclear graphite. Even though ion irradiation is thought of as a neutron surrogate, their damage volumes and rates are dramatically different. Neutrons damage graphite very differently from the ions generated by a linear accelerator because they are slower and chargeless compared to the ions. Future studies should include different sets of experiments for nano, micro, and meso scales and consider the comprehensive design of experiments in terms of irradiation energy and fluence. The impact of these studies will be improved if these studies can be performed at higher temperatures and neutron irradiation conditions relevant to nuclear reactors.

## 5. Conclusions

The creep deformation of nuclear graphite is a critical parameter in the operation and longevity of reactors because it plays both desirable and undesirable roles. Even though mathematical models backed up with experimental results are available at the bulk scale, a close view at the atomic scale to nanoscale is important to understand the fundamental mechanisms behind deformation in this brittle material. This study aims to achieve this with in situ TEM creep experiments, noting that the current literature only performs postmortem or basic imaging analysis with TEM. However, real-time creep loading and simultaneous imaging are essential to capture the essence of dynamic deformation. Micro-pillar specimens were prepared and evaluated with specified peak loads, loading and unloading times, and holding times inside a TEM. This was followed by indentation creep experiments. The following conclusions can be made from the experimental results:

(a) Even though the bulk nuclear graphite is very brittle, there are nanoscale regions that are extremely active in terms of dislocation generation and motion under both increasing and constant load. These are ligaments of bands with widths corresponding to the distance between two Mrozowski cracks. They contain dense dislocation networks even at no load.
(b) At very high load levels, deformation is activated in regions with no pre-existing defects but with the highest stress concentrations.
(c) The role of ion irradiation is to increase point defects and clusters (depending on the energy and fluence) in the specimen. Smaller clusters from self-ion irradiation are observed to make the specimen more brittle, while very large clusters from heavy ion irradiation can show the opposite behavior.
(d) The deformation bands are highly localized in the brittle matrix material that shows no indications of deformation mechanisms. The localization phenomenon is therefore influenced by the pre-existing defects (e.g., Mrozowski cracks). The localization also leads to a deformation-band-type geometry for plasticity, like kink or ripplocation bands.

The inherent heterogeneity in material composition and defect length scale makes quantitative study very difficult. The in situ TEM experiments are therefore important for visual cues regarding only the creep deformation in the material. Future studies should involve high-temperature and neutron irradiation relevant to nuclear reactors. Also important is to study intermediate-length-scale specimens (micron-scale thickness). This is not possible with TEM, but other techniques (e.g., X-ray computed tomography) could be helpful to connect the nanoscale mechanisms to the bulk behavior.

## Data Availability

Data can be obtained by email request.

## Supplementary Materials

The following supporting information can be downloaded at: https://www.mdpi.com/article/10.3390/ma17184530/s1, Information on TEM specimen preparation and in situ TEM experiment videos are available as Supporting Information embedded in PPT format for cross-platform access.
