# Supplementary material for: Dynamic Deformation in Nuclear Graphite and Underlying Mechanisms
Source: Materials (Basel). 2024 Sep 14;17(18):4530. doi: 10.3390/ma17184530 (PMC11433592; doi:10.3390/ma17184530)
Supplement: Supplementary file 1 [file materials-17-04530-s001.zip › materials-3200221-supplementary.pdf]

Supporting Information

Dynamic Deformation in Nuclear Graphite and Fundamental Mechanisms

Melonie Thomas [1], Hajin Oh [1], Ryan Schoell [2], Stephen House [2], Miguel Crespillo [3], Khalid Hattar [3], William Windes [4] and Aman Haque [1,*]

[1] Department of Mechanical Engineering, The Pennsylvania State University, University Park, PA 16802, USA; melonie.thms@gmail.com (M.T.); oh339@purdue.edu (H.O.)
[2] Center for Integrated Nanotechnologies, Sandia National Laboratories, Albuquerque, NM 87185, USA; rmschoe@sandia.gov (R.S.); sdhouse@sandia.gov (S.H.)
[3] Department of Nuclear Engineering, University of Tennessee, Knoxville, TN 37996, USA; mcrespil@utk.edu (M.C.); khattar@utk.edu (K.H.)
[4] Idaho National Laboratory, Idaho Falls, ID 83415, USA; william.windes@inl.gov
* Correspondence: mah37@psu.edu; Tel.: +1-814-865-4248

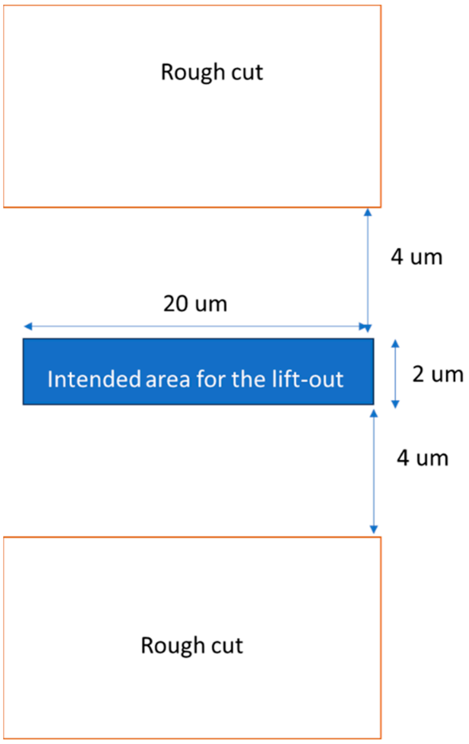

Figure S1. The first step in the FIB procedure designed to minimize ion beam damage to the sample area.

The rough cut is performed with a 4 um distance from the intended lamella area from each side to minimize Ga ion beam damage to the sample. The fine cuts were then performed at angle of 52±5° (front and back sides) starting from 3 nA and reducing to 1 nA as the “intended area for the lift-out” approaches.

Table S1. Beam current, angle, and thickness parameters for the FIB fabrication of micropillars

Ion beam current	Tilt angle (°)	Thickness after milling	Location of the specimen
15 nA	0	10 um	Trench/bulk
3 nA	57 (front) and 47 (back)	4 um	Trench/bulk
1 nA	57 (front) and 47 (back)	1 um	Trench/bulk
0.5 nA	54 (front) and 50 (back)	800 nm	Grid
0.1 nA	54 (front) and 50 (back)	400 nm	Grid
50 pA	54 (front) and 50 (back)	160 nm or final thickness	Grid
50 PA	-14	Pillars	Grid

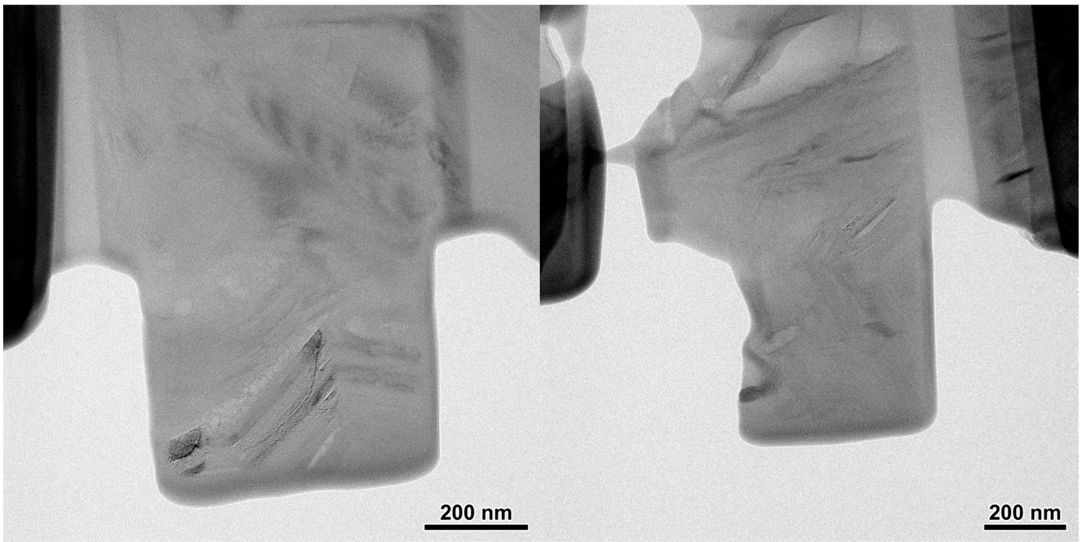

Figure S2 TEM micrographs of a pristine specimen fabricated into micropillars. Electron transparent specimens comprise of Filler particle regions, amorphous binder areas, and Mrozowski cracks, and are visible in micrographs.
