# Supplementary material for: Dynamic Deformation in Nuclear Graphite and Underlying Mechanisms
Source: Materials (Basel). 2024 Sep 14;17(18):4530. doi: 10.3390/ma17184530 (PMC11433592; doi:10.3390/ma17184530)
Supplement: Supplementary file 1 [file materials-17-04530-s001.zip › Supplementary_Video.pptx]

## Slide 1
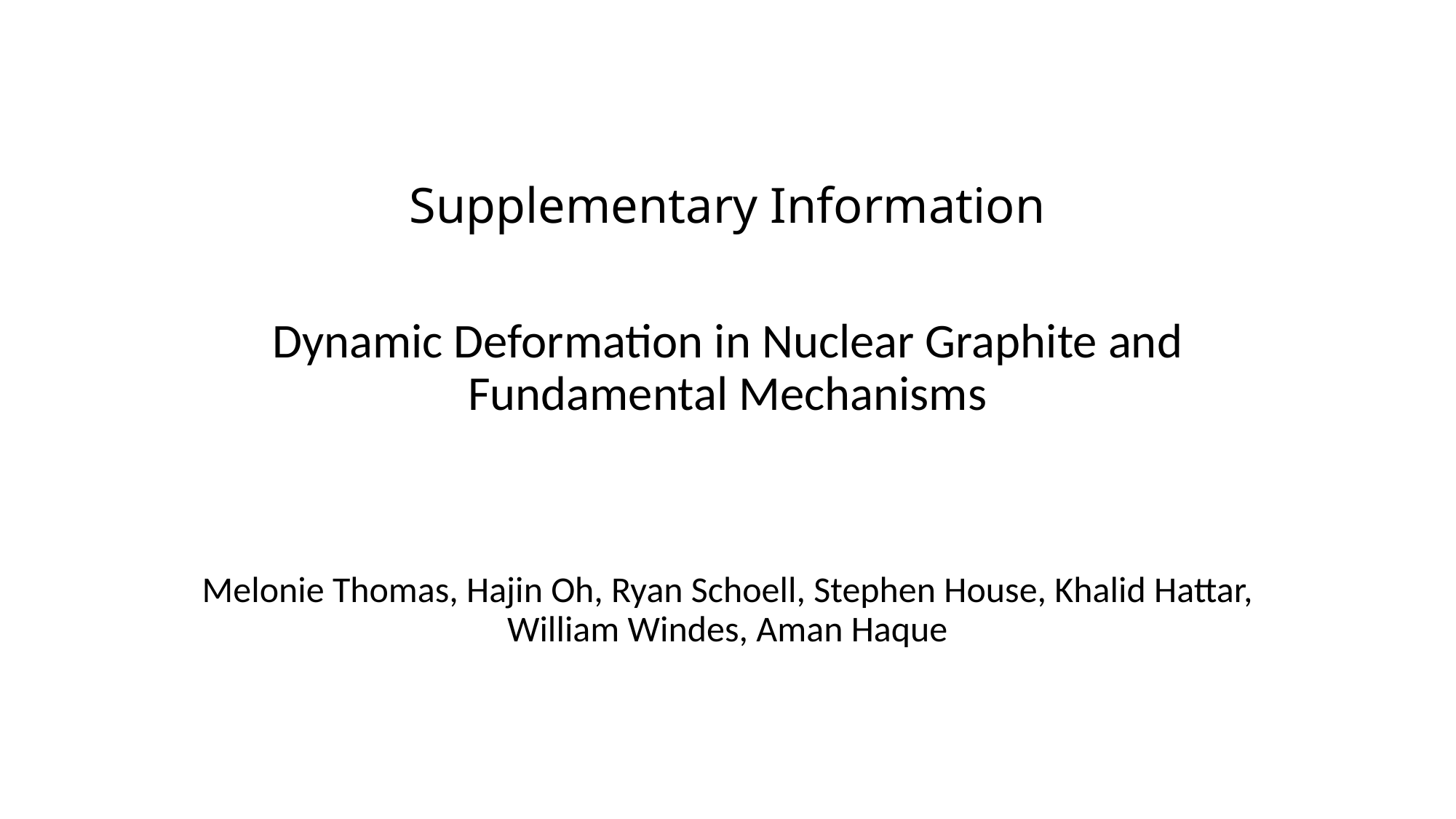

# Supplementary Information
Dynamic Deformation in Nuclear Graphite and Fundamental Mechanisms
Melonie Thomas, Hajin Oh, Ryan Schoell, Stephen House, Khalid Hattar, William Windes, Aman Haque

## Slide 2
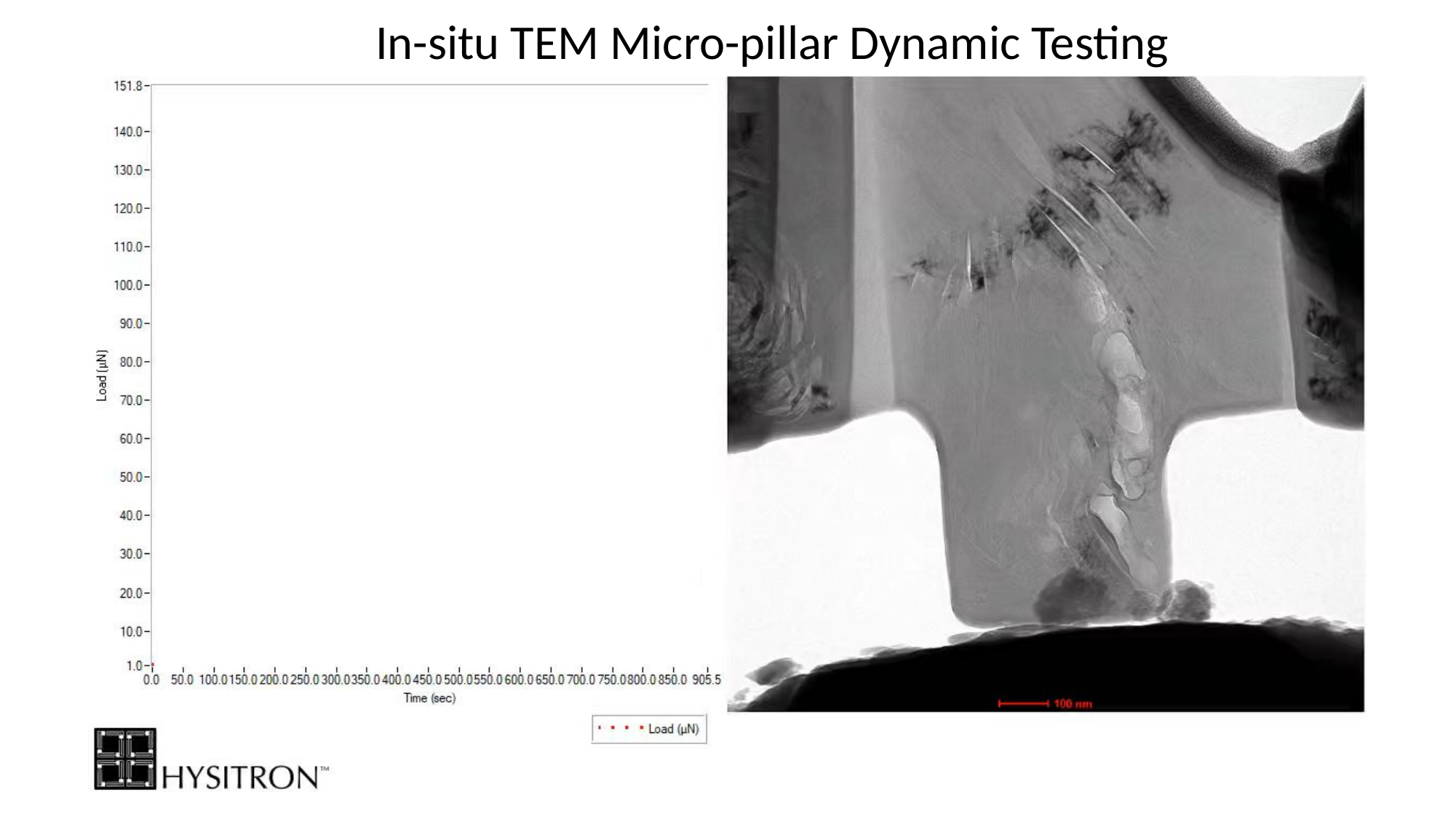

In-situ TEM Micro-pillar Dynamic Testing
